# Optimizing neurogenic differentiation of dental stem cells: a critical review of induction strategies and functional validation

**DOI:** 10.3389/fdmed.2026.1955032

**Published:** 2026-09-21

**Authors:** Masar Mohsin Alsultani, Anas Falah Mahdee

**Affiliations:** 1 Department of Restorative and Aesthetic Dentistry, College of Dentistry, University of Babylon, Babylon, Iraq; 2 Department of Restorative and Aesthetic Dentistry, College of Dentistry, University of Baghdad, Baghdad, Iraq

**Keywords:** dental stem cells, electrophysiology, growth factors, neurogenesis, small molecules

## Abstract

Neurogenesis is highly active during brain development but becomes substantially restricted in adulthood. Therefore, the nervous system shows a limited ability to self-renew succeeding a traumatic incident or neurodegenerative disease. Dental stem cells (DSCs) represent a unique population of mesenchymal stem cells originating from the cranial neural crest. Thus, they have an inherent capacity to differentiate into neurons under proper *in vitro* cues. However, this ability is limited by low conversion rates and unpredictable target cell types, along with a noticeable lack of standardized differentiation protocols. Therefore, the primary aim of this critical narrative review is to evaluate, categorize, and critique the neurogenic induction strategies for human dental stem cells reported over the last ten years. By analyzing the current literature, differentiation protocols can be broadly categorized into three distinct approaches: growth factors, small molecules, and combination of both. Although the combined strategy shows a promising potential, the focus on structural outcomes and the absence of electrophysiological validation make it hard to determine which protocol truly generates functional neurons. Moreover, in the few protocols that provided differentiation evidence across morphological, molecular, and functional endpoints, the resulting cells displayed only partial functional maturation, indicating that the generation of fully mature neurons remains challenging *in vitro*. Moving forward, future studies should incorporate patch-clamp electrophysiology as a standard component of functional validation to determine whether differentiated cells acquire genuine neuronal excitability and to strengthen the evidence required for clinical translation.

## 1 Introduction

The process of neurogenesis is governed by neural stem cells (NSCs), a specialized population of multipotent cells with the ability to differentiate into both neuronal and glial lineages. While it is a natural process during development, neurogenesis sharply declines in adulthood (1). Therefore, after a traumatic event or neurodegenerative disease, the nervous system exhibits a limited capacity for self-renewal (2).

A similar process arises in dental tissues, in which severe inflammation often causes irreversible damage to the blood vessels and nerves in the pulp. Since irreversibly inflamed pulp is incapable of self-repair, modern regenerative endodontics aims for functional pulp regeneration, including dental nerves. Re-establishing this neural network is essential for regulating pulpal blood flow and immune defenses, and for driving the overall reparative process (3–5).

Consequently, researchers are increasingly exploring *in vitro* differentiated stem cells as a prospective therapeutic approach for *in vivo* nerve regeneration (6). Because endogenous adult NSCs are scarce and difficult to harvest, the field has shifted toward identifying alternative stem cell sources for neuro-regenerative therapies (7).

While embryonic stem cells were the first to be directed into specific lineages, their clinical application remains restricted by biological and ethical concerns (8). Mesenchymal stem cells (MSCs), which can be found in nearly all postnatal tissues, offer a practical alternative. Under appropriate environmental cues, MSCs can differentiate into various mesodermal lineages such as osteocytes, adipocytes, and chondrocytes (9).

However, a unique subtype of MSCs known as dental stem cells (DSCs) shows broader plasticity. Owing to their neural crest origin, DSCs can additionally differentiate into ectodermal lineages, including both neuronal and neuroglial cells (10). Several types of DSCs have been successfully isolated and categorized based on their specific tissue of origin, with the most prominent being dental pulp stem cells (DPSCs), stem cells from the apical papilla (SCAPs), and stem cells from human exfoliated deciduous teeth (SHEDs) (11). Compared to other mesenchymal populations, DSCs inherently possess neuron-like characteristics, granting them a higher potential for *in vitro* induction into functional neurons (12). However, these cells have specific limitations, mainly a poor differentiation rates and inconsistent lineage specification (13).

Cellular differentiation is governed by both intrinsic factors and extracellular environment. It is classically regulated *ex vivo* by cell culture modulation with cocktails of growth factors, signaling molecules, and/or genetic manipulation (14). By controlling these factors, stem cells can be directed toward a specialized, physiologically active fate that potentially helps to restore tissue integrity and functionality (15). Unfortunately, the literature reveals a notable lack of standardized and functionally optimized differentiation protocols that are used to obtain mature and fully differentiated neurons. While some reviews have discussed the neurogenesis of dental stem cells, their primary focus was on stem cell biology, cellular heterogeneity, and broad summaries of *in vitro* methodologies (12), they often lack a structured categorization of induction strategies. This review offers an organized comparison of three induction approaches, including growth factors, small molecules, and their combination. Furthermore, it uniquely critiques the functional validation of these approaches, moving beyond basic structural phenotypes to evaluate electrophysiological maturity. Therefore, the primary aim of this review is to summarize, categorize, and criticize the neurogenic differentiation protocols for human dental stem cells that have been published over the past decade. This can help researchers choose the most appropriate evidence-based neuronal differentiation route to be followed in tissue regeneration.

## 2 Methodology

To structure this critical narrative review and minimize selection bias, a targeted literature search was conducted in the PubMed database to detect related English-language articles published from January 2016 to April 2026. The search terms involved combinations of dental stem cell, neurogenic outcome, and common neurogenic inducers. The keyword combination structured as follows: (“dental-derived stem cells” OR “dental stem cells” OR “human dental pulp stem cells” OR “stem cells from apical papilla” OR “stem cells from human exfoliated deciduous teeth”) AND (“neurogenic differentiation” OR “neural induction” OR “neurogenic potential” OR “neurogenesis” OR “neurosphere” OR “neuron-like cells”) AND (“differentiation protocol” OR “differentiation medium” OR “protocol” OR “induction medium” OR “small molecules” OR “cocktail” OR “resveratrol” OR “retinoic acid” OR “Forskolin” OR “growth factor” OR “BDNF” OR “EGF” OR “bFGF” OR “NGF” OR “commercial medium”) NOT (“clinical trials” OR “review”).

### 2.1 Inclusion and exclusion criteria

The review included original research articles (both *in vitro* and *in vivo* studies) that presented outcomes meeting our predefined criteria for neuronal differentiation of human dental stem cells, specifically the validation of neural phenotypes through protein-level or functional evidence, thereby excluding studies that relied solely on transcriptional analysis. Studies utilizing specific chemical inducers (such as *β*-mercaptoethanol and butylated hydroxyanisole) as standalone agents were also excluded, as these are known to induce false-positive artifactual morphological changes rather than true functional neurogenesis. Clinical trials, narrative or systematic reviews and protocol articles were likewise excluded. After initial research, 83 articles were identified, after excluding duplicates and skimming titles and abstracts for relevant neurogenic studies, only 50 articles remained. These were further evaluated by full-text reading and only 25 articles met the inclusion criteria. Three relevant papers were manually added through screening the reference lists of the retrieved articles, as their specific keywords were not captured by the primary search string. These studies were evaluated for the same inclusion and exclusion criteria applied to the primary database search. In total, 28 studies were included in the final structure.

## 3 Extracted data

Various protocols have been developed to induce neuronal differentiation of DSCs. Within the scope of the reviewed literature, 31 differentiation protocols were identified and broadly categorized into three distinct approaches (see Table 1). The first approach (8 protocols) relies primarily on exogenous growth factor supplementation such as epidermal growth factor (EGF), basic fibroblast growth factor (bFGF), and brain-derived neurotrophic factor (BDNF). The second (4 protocols) utilizes neurogenic small molecules and chemical inducers (e.g., forskolin, tricyclodecane-9-yl-xanthogenate). While the majority of the selected studies (19 protocols) employed a combined strategy, utilizing both growth factors and small molecules to stimulate the innate neurogenic capacity of DSCs. In addition, the type of dental stem cell used, cell culture type, basal and differentiation media, induction period, and differentiation outcomes were all identified and summarized in Table 1.

**Table 1:** Summary of neurogenic differentiation protocols for human dental stem cells and their outcomes.

Induction strategy	Study	Human stem cell type	Culture type/ Coating	Basal media	Neurogenic cocktail	Induction period	Target cell type	Phenotype validation	Biomarker detection assays	Structural/ functional validation
Growth factor-mediated differentiation	Madanagopal et al. (18)	DPSC	2D adherent monolayer/ uncoated.	Neurobasal-A medium +B27 supplement (xeno free).	20 ng/ml EGF +40 ng/ml bFGF.	21 days.	Pan-neuronal	NF-H	qRT-PCR FCM	Not assessed.
	Farhang et al. (21)	DPSC	2D adherent monolayer/ uncoated.	Presumed Neurobasal™ medium +10% FCS based on their parallel 3D protocol.	Phase 1 (5 days): 100 ng/ml bFGF. Phase 2 (5 days): 100 ng/ml SHH.	10 days.	Pan-neuronal	NeuN	ICC/IF	Not assessed.
			3D hanging drop dissociated to 2D adherent monolayer.	Neurobasal™ medium +10% FCS.	Phase 1 (Preconditioning/2 days): 20 ng/ml EGF +40 ng/ml bFGF. Phase 2 (5 days): 100 ng/ml bFGF. Phase 3 (5 days):100 ng/ml SHH.	12 days.	Pan-neuronal	NeuN	ICC/IF	Not assessed.
	Singh et al. (22)	DPSC	2D adherent monolayer/ uncoated.	Neurobasal™ medium +B27 supplement + L-Glutamine (serum free).	Phase 1 (8 days): 10 ng/ml EGF +10 ng/ml FGF2. Phase 2 (4 days): Addition of 50 ng/ml BDNF.	12 days.	Dopaminergic neuron	MAP2 TH	qRT-PCR ICC/IF FCM WB	Calcium imaging (+).
	Kawase-Koga et al. (20)	DPSC	3D neurosphere transferred to 2D adherent monolayer (Poly-L-lysine + Laminin).	Phase1: DMEM/F12 (xeno-free) Phase2: DMEM/F12 + 1% FBS.	Phase1(Neurosphere/ 7 days): 20 ng/ml EGF +10 ng/ml bFGF + B27 + N2). Phase 2: (Maturation/ 21 days): N2 + B27.	28 days.	Pan-neuronal	NF-M	qRT-PCR ICC/IF	Not assessed.
Growth factor-mediated differentiation	Bojnordi et al. (23)	DPSC	3D neurosphere transferred to 2D adherent monolayer (Poly-L-lysine coated).	Neurobasal™ medium (serum free).	Phase 1(Neurosphere/ 10 days): 20 ng/ml EGF +10 ng/ml bFGF + B27). Phase 2: (Maturation/ 10 days): N2 + B27.	20 days.	Pan-neuronal	MAP2	ICC/IF	Not assessed.
	Luzuriaga et al. (24)	DPSC	3D dentosphere transferred to 2D monolayer (Laminin/Poly-lysine coated).	phase 1: StemPro™ MSC SFM basal medium + StemPro™ MSC SFM supplement (9:1 ratio). phase 2: Neurocult™ Differentiation Medium (serum free).	Phase 1(Preconditioning/ 14 days): 500 ng/ml BDNF +20 ng/ml NT-3. Phase 2 (Differentiation/ 21 days): 20 ng/ml EGF +10 ng/ml bFGF +2% B27 +2 µg/ml heparin.	35 days.	Dopaminergic neuron	NeuN MAP2 TH	ICC/IF	Not assessed.
	Li et al. (25)	DPSC	Electrospun Scaffold (Heparin-modified gelatin/polyc-aprolactone nanofiber membrane).	*α*-MEM +10% FBS.	Sustained release via scaffold: 100 ng/ml VEGF/NGF +50 ng/ml BMP-2.	7 days *in vitro* +12 weeks *in vivo*	Pan-neuronal	NeuN	qRT-PCR WB	*In vivo* (Subcutaneous root implant): Histology & IHC confirmed successful neural and vascular ingrowth.
Small molecule- mediated differentiation	Baysal et al. (29)	DPSC (Commercial)	2D adherent monolayer/ Cell-matrix basement membrane gel coated.	Commercial dopaminergic differentiation medium (serum free).	10 μM melatonin.	21 days.	Dopaminergic neuron	NeuN TH	ICC/IF WB	Not assessed.
	Jang et al. (30)	DPSC (Cryo-preserved)	2D adherent monolayer/Geltrex™ -coated.	ADMEM +10% FBS.	4 µg/mL tricyclodecane-9-yl-xanthogenate (D609).	3 days.	cholinergic neurons	ChAT HB9 ISL1	qRT-PCR ICC/IF WB	*In vitro*: Neurotransmitter release (Acetylcholine) (+). *In vivo*: Open-field test (+).
	Kang et al. (31)	DPSC	2D adherent monolayer/ uncoated.	ADMEM (serum-free).	15 µg/mL tricyclodecane-9-yl-xanthogenate (D609).	4 days.	cholinergic neurons	ChAT HB9 ISL1 MAP2	qRT-PCR ICC/IF	Neurotransmitter release (Acetylcholine) (+).
	Pardo-Rodríguez et al. (33)	DPSC	3D dentosphere transferred to 2D adherent monolayer (Laminin coated).	Neurocult™ basal medium +2% B27 (with vitamin A) (serum free).	Neurocult™ differentiation supplement +10 µM RA + 40 mM KCl pulses (1 h every 2 days starting at day 7).	21–60 days.	Pan-neuronal	MAP2 NeuN SynapsinPSD95 GAD65	qRT-PCR ICC/IF	patch-clamp: voltage-clamp mode (+), current-clamp mode (+).
Combination- mediated differentiation	Goudarzi et al. (35)	DPSC	3D neurospheres transferred to 2D adherent monolayer (uncoated).	Phase 1: DMEM/F12 (serum-free). Phase 2: DMEM/F12 +5% FBS.	Phase 1(Neurosphere/ 3 days): 5% CSF. Phase 2: (Maturation/ 5 days): N2 + 2% B27 +10^−7^μM RA +100 ng/ml GDNF +200 ng/ml BDNF.	8 days.	Pan-neuronal	MAP2	ICC/IF	Nissl bodies staining (+).
	Haratizadeh et al. (40)	DPSC	2D adherent monolayer/ uncoated.	DMEM/F12 (serum free) (Presumed from expansion phase).	10^−6^ μM RA +10% CSF.	8 days.	Pan-neuronal	MAP2	ICC/IF	Nissl bodies staining (+).
	Kogo et al. (41)	DPSC	2D adherent monolayer/ uncoated.	DMEM/F12	Phase 1 (2 days): 2.5% FBS +10 μM Azacytidine +10 ng/ml bFGF. Phase 2 (20 min): High K^+^ BSS-Ca^+2^ buffer. Phase 3 (3 days): 1% insulin-transferrin-selenium +250 μM 3-isobutyl-1methylxanthine +50 μM forskolin + 1 mM dbcAMP +200 nM tissue plasminogen activator +10 ng/mL NGF +30 ng/mL NT-3 + 10 ng/mL bFGF.	5 days.	Pan-neuronal	PSD95.	ICC/IF	Not assessed.
Combination- mediated differentiation	Cui et al. (42)	SCAP ^a^	2D adherent monolayer/ uncoated.	DMEM.	Phase 1 (24 h): 20% FBS +10 ng/mL bFGF. Phase 2 (3 days): 2% DMSO +200 μM BHA +25 mM KCl +10 μM hydrocortisone +5 μg/ml insulin +2 mM valproic acid.	4 days.	Pan-neuronal	GAD67 vGLUT1	ICC/IF	Nissl bodies staining (+).
	Zou et al. (44)	SCAP	3D hydrogel encapsulation.	DMEM/F12: Neurobasal-A medium [1:1] + 1% N2 + 2% B27 (serum-free).	Phase 1 (Preinduction/7 days): 100 μM cAMP + and 20 ng/ml bFGF. Phase 2 (Induction/ 3–7 days): matrix immersed in 100 μM cAMP +20 ng/ml bFGF +20 ng/ml BDNF +20 ng/ml GDNF +20 ng/ml NT3 +3 μM CHIR99021 +10 μM Forskolin +1 μM Dorsomorphin.	10–14 days.	Pan-neuronal	MAP2	qRT-PCR ICC/IF WB	Not assessed.
	Messat et al. (43)	DPSC	3D neurospheres (ultra-low attachment plate) transferred to 2D monolayer (Geltrex coated).	DMEM/F12 (serum free).	Phase 1 (Neurosphere formation/ 7 days): 1% N2 + 1% B27 +20 ng/mL EGF +20 ng/mL bFGF. (the first 3 days media supplemented with 1 µM SB4,31,542 and 1 µM LDN-1,93,189 and for the last 4 days with 1 µM SB431542 and 10 ng/mL BMP4). Phase 2 (Maturation/ 14 days): 1% N2 + 1% B27 + 20 ng/mL BDNF +30 ng/mL NT3.	21–32 days.	SGN	NeuN MAP2 BRN3APRPH TRKC	ICC/IF	AFM-based nanomechanical characterization (+): Young's modulus measurements of SGN *in vivo* and *in vitro* were similar. ICC/IF (+): SGN from newborn rats send out neurites that contact DPSC-derived ONPs.
Combination- mediated differentiation	Geng et al. (13)	DPSC	2D adherent monolayer/ uncoated.	DMEM/F12.	phase1 (pretreatment/12 h): 15 μM resveratrol. phase 2 (24 h): 10% FBS +10 ng/ml bFGF +500 μM *β*-mercaptoethanol. phase 3 (6 h): 100 μM BHA +2% DMSO (serum-free).	42 h.	Pan-neuronal	NFM	ICC/IF WB	Not assessed.
	Songsaad et al. (46)	SCAP	2D adherent monolayer/ poly-d-lysine coating.	DMEM/F-12	Phase 1: (pretreatment/12 h): 10 μM resveratrol. Phase 2 (24 h): 10% FBS +10 ng/ml bFGF +500 μM β-mercaptoethanol. Phase 3 (6 h): 100 μM BHA +2% DMSO (serum-free).	42 h.	Pan-neuronal	NF MAP2	PCR ICC/IF	calcium imaging (+). Nissl bodies staining (+).
	Heng et al. (27)	DPSC SCAP GMSCs	2D adherent monolayer/ uncoated	DMEM/F12: Neurobasal (1:1) + 0.5% N2 + 1% B27 (Serum-Free)	Phase 1 (8 days): 20 ng/mL bFGF +0.5 mM VPA +3 μM CHIR99021 +1 μM Repsox +10 μM Forskolin +10 μM SP600125 +5 μM GO6983 +5 μM Y-27632 +100 μM cAMP. Phase 2 (6 days): 20 ng/mL (bFGF +BDND + GDNF+NT3) +3 μM CHIR99021 + 10 μM Forskolin +1 μM Dorsomorphin +100 μM cAMP.	14 days.	Pan-neuronal	NeuN MAP2	qRT-PCR ICC/IF	Calcium imaging (+).
	Thongsuk et al. (48)	DPSC	2D adherent monolayer/ uncoated.	DMEM/F-12 + 10% FBS.	Phase 1 (24 h): 10 ng/ml bFGF +500 μM beta-mercaptoethanol + ergothioneine. Phase 2 (6 h): 2% DMSO +100 μM BHA + ergothioneine.	30 h.	Pan-neuronal	MAP2 SV2A	qRT-PCR ICC/IF	Calcium imaging (+). Nissl bodies staining (+).
Combination- mediated differentiation	Singh et al. (51)	DPSC	2D adherent monolayer/ uncoated.	Neurobasal + B27 (serum free).	10 ng/ml EGF +50 ng/ml FGF2 +10 μM Forskolin.	14 days.	Dopaminergic neuron	MAP2 TH DAT synaptophysin	qRT-PCR ICC/IF FCM	Calcium imaging (+). Neurotransmitter release (Dopamine) (+). SEM/TEM- based ultra-structural level (+).
	Kang et al. (31)	DPSC	2D adherent monolayer/ uncoated	ADMEM (Serum-Free)	Phase 1 (24 h): 1 mM β-mercaptoethanol. Phase 2 (6 days): 100 ng/ml NGF	7 days.	Cholinergic neurons	ChAT HB9 ISL1 MAP2 BETA-3.	qRT-PCR ICC/IF	Neurotransmitter release (Acetylcholine) (+)
			2D adherent monolayer/ uncoated	ADMEM (Serum-Free)	10 ng/ml bFGF +50 µM Forskolin +250 ng/ml SHH +0.5 µM RA	7 days.	Cholinergic neurons	ChAT HB9 ISL1 MAP2 BETA-3.	qRT-PCR ICC/IF	Neurotransmitter release (Acetylcholine) (+)
	Al-Maswary et al. (4)	DPSC	2D adherent monolayer (collagen-I coated).	DMEM/F12.	Phase 1 (5 days): 10% FBS +10 μM ATRA. Phase 2 (7 days): 50 ng/ml BDNF (serum-free)	12 days.	sensory cholinergic neuron	NF-M	qRT-PCR ICC/IF	patch-clamp: voltage-clamp mode (+).
Combination- mediated differentiation	Li et al. (52)	DPSC SCAP GMSCs	2D monolayer/ uncoated.	α-MEM (serum-free)	10 ng/ml bFGF +10 μM Forskolin +25 mM KCl +2 mM valproic acid +5 μg/ml insulin.	35 days.	Pan-neuronal	NFM	qRT-PCR ICC/IF	patch-clamp: voltage-clamp mode (−), current-clamp mode (−). (Zero electrical currents and no action potentials).
		DPSC GMSCs	3D neurospheres (ultra-low attachment Petri dishes) transferred to 2D adherent monolayer (poly-l-ornithine/laminin coated).	phase 1: DMEM/F12 + 2% B27 (serum free). phase 2: Neurobasal medium +2 mM L-glutamine +2% B27 + 1% N2 (serum free).	Phase 1 (Neurosphere formation/ 1 week): 20 ng/ml EGF +20 ng/ml bFGF. Phase 2 (maturation/4 weeks): 1 mM dbcAMP +30 ng/mL NT-3.	5 weeks.	Pan-neuronal	MAP2 NFM	qRT-PCR ICC/IF	patch-clamp: voltage-clamp mode (+), current-clamp mode (+).
	Chen et al. (53)	SCAP	2D adherent monolayer/ uncoated.	Neurobasal-A + DMEM/F12 (1:1) + 0.5% N2 + 1% B27 (serum-free).	100 mM cAMP +20 ng/ml bFGF +10 μM Forskolin +0.5 mM valproic acid +3 μM CHIR99021 + 1 μM Repsox +10 μM SP600125 + 5 μM GO6983 + 5 μM Y-27632.	7 days.	Pan-neuronal	NF-M NeuN MAP2	qRT-PCR WB	patch-clamp: voltage-clamp mode (+), current-clamp mode (+).
Combination- mediated differentiation	Koh et al. (54)	SCAP	2D adherent monolayer/ uncoated.	Neurobasal-A + DMEM/F12 (1:1) + 0.5% N2 + 1% B27 (serum-free).	100 mM cAMP +20 ng/ml bFGF +10 μM Forskolin +0.5 mM valproic acid +3 μM CHIR99021 + 1μM Repsox +10 μM SP600125 + 5 μM GO6983 +5 μM Y-27632.	7 days.	Pan-neuronal	NeuN NF-M MAP2	ICC/IF	*in vivo* IF + myelinated fiber count (−): Poor structural integration. Walking track analysis (+). Toe spread test (−).
	Zhang et al. (55)	SHED	2D adherent monolayer/ Poly-ornithine and laminin.	DMEM/F12 + N2 supplement (serum free).	Phase 1 (7 days):10 ng/ml FGF2/EGF/FGF8 +100 ng/ml SHH + Noggin. Phase 2 (7 days): 100 ng/ml SHH +10 ng/ml FGF8 + CHIR99021 + 200 mmol/L AAP. Phase 3 (7 days): 50 ng/ml BDNF/GDNF + TGF-β +200 mmol/L AAP.	21 days.	Dopaminergic neuron	*In vitro*: MAP2 TH DAT *in vivo*: TH DAT	*In vitro*: qRT-PCR, ICC/IF *in vivo*: IHC/IF	*In vitro* patch-clamp: voltage-clamp mode (+), current-clamp mode (+). *In vivo*: Amperometric recording (+); rotation test (+): reduced motor asymmetry; rotarod test (+): improved balance.

This table provides a comprehensive summary of neurogenic differentiation strategies applied to human dental stem cells, categorizing them into growth factor-mediated, small molecule-mediated, and combination-mediated approaches. It details the specific experimental parameters for various studies, outlining the human stem cell type, culture type and coating, basal media, and the specific neurogenic cocktail used for each protocol. Additionally, the table specifies the required induction period and the ultimate target cell type for each method. Finally, it summarizes the phenotype validation techniques employed, reporting the specific biomarker detection assays alongside the methods used for structural and functional validation, such as calcium imaging, patch-clamp recordings, and *in vivo* testing.

2D, two-dimensional; 3D, three-dimensional; α-MEM, minimum essential medium-alpha; AAP, ascorbic acid 2-phosphate; ADMEM, advanced Dulbecco's modified Eagle medium; ATRA, all trans retinoic acid; AFM, atomic force microscopy; BDNF, brain-derived neurotrophic factor; bFGF, basic fibroblast growth factor; BSS, balanced salt solution; BMP-2, bone morphogenetic protein 2; BRN3, brain-3; BHA, butylated hydroxyanisole; ChAT, choline acetyltransferase; CSF, cerebrospinal fluid; cAMP, cyclic adenosine monophosphate; D609, tricyclodecane-9-yl-xanthogenate; DAT, dopamine transporter; dbcAMP, dibutyryl cyclic adenosine monophosphate; DMSO, dimethyl sulfoxide; DMEM/F12, Dulbecco's modified Eagle medium/F12; DPSC, dental pulp stem cell; EGF, epidermal growth factor; FBS, fetal bovine serum; FCM, flow cytometry; FCS, fetal calf serum; FGF2, fibroblast growth factor 2; FGF8, fibroblast growth factor 8; GAD65, glutamic acid decarboxylase 65; GDNF, glial cell line-derived neurotrophic factor; GMSC, gingival mesenchymal stem cell; HB9, homeobox protein HB9; ICC, immunocytochemistry; IF, immunofluorescence; IHC, immunohistochemistry; ISL1, ISL LIM homeobox 1; KCl, potassium chloride; MAP2, microtubule-associated protein 2; MSC, mesenchymal stem cell; NeuN, neuronal nuclei; NF-H, neurofilament heavy chain; NF-M, neurofilament medium chain; NGF, nerve growth factor; NT-3, neurotrophin-3; ONP, otic neural progenitor; PSD95, postsynaptic density protein 95; PRPH, peripherin; qRT-PCR, quantitative reverse transcription polymerase chain reaction; RA, retinoic acid; SCAP, stem cell from the apical papilla; SEM, scanning electron microscopy; SGN, spiral ganglion neuron; SHED, stem cells from human exfoliated deciduous teeth; SHH, sonic hedgehog; SFM, serum-free medium; SV2A, synaptic vesicle glycoprotein 2A; TEM, transmission electron microscopy; TRKC, tropomyosin receptor kinase C; TGF-β, transforming growth factor beta; TH, tyrosine hydroxylase; VEGF, vascular endothelial growth factor; vGLUT1, vesicular glutamate transporter 1; WB, western blotting; (+): positive detection or successful validation; (−): negative detection or lack of functional activity;.

a Lentivirus-induced SCAP overexpression of insulin-like growth factor.

### 3.1 Growth factor-mediated neurogenic induction

The differentiation of neural stem cells is a highly complex process that relies heavily on the modulatory effects of diverse growth factors. To replicate the developmental cues of the embryonic nervous system, exogenous growth factors and neurotrophins are classically used *in vitro* to stimulate neurogenesis and guide stem cell fate (16). Upon ligand-receptor engagement, downstream signaling cascades are triggered to dictate targeted gene expression. These transcriptional changes subsequently alter cellular metabolism, protein production, ion dynamics, and cytoskeletal architecture, collectively orchestrating the cell's final phenotypic transformation (17).

Among the growth factor-based protocols, some studies employed 2D adherent monolayer culture in all differentiation phases. In one approach, a 21-day, single-phase protocol cultured DPSCs in a xeno-free, bFGF/EGF-based medium to induce neuronal morphology and mature neural proteins while downregulating stemness markers (18). Using serum-free differentiation media has major advantages for future clinical translation, this strategy avoids xenogenic substances that may cause graft rejection, host immune reactivity, or contamination (19, 20).

Taking a multiphase approach, Farhang, Soleimani (21) reported that using a two-step strategy, by first exposing DPSCs to bFGF for five days, followed by a second five-day exposure to the developmental morphogen sonic hedgehog (SHH), yielded significantly more neuronal nuclei (NeuN)-positive cells than bFGF alone. This is likely because SHH triggers a broader neurogenic signaling cascade.

Another study reported that adding BDNF to a baseline EGF/FGF2 culture for 12 days upregulated mature structural and dopaminergic markers. In addition, by employing a more advanced assessment, they confirmed the presence of voltage-gated ion channels using intracellular calcium imaging (22).

While many studies used calcium imaging to demonstrate functional maturation, it is important to note that the observed calcium influx is often due to forced chemical depolarization by exposure to concentrated potassium chloride (KCl) solution rather than spontaneous network activity. Furthermore, voltage-gated calcium channels are specialized membrane proteins found across many eukaryotic cell types, not exclusively in neurons. Hence, calcium imaging confirms the presence of working ion channels in response to stimuli, yet it remains an indirect measure of true electrical firing.

Moving beyond flat monolayers, half of the evaluated growth factor protocols incorporated a 3D neurosphere formation step before prompting neurogenesis. Culturing cells in 3D mimics the floating nature of neural stem cells in their niches, which could offer improved intercellular interactions, promoting a more complete differentiation process (21). Applying this strategy, Farhang, Soleimani (21) directly compared the efficacy of their previously mentioned 2D induction protocol against a 3D model. By adding a preconditioning 48 h neurosphere formation step to the same bFGF/SHH protocol, they found that the 3D model showed higher expression of neuronal markers compared to the 2D.

Two separate studies utilized a nearly identical two-phase 3D cultivation method (20, 23). In the first phase, stem cells were cultured in a serum-free basal medium containing EGF and bFGF to form neurospheres. In the second phase, they triggered terminal neural differentiation by intentionally removing these growth factors from the medium. Both studies showed structural neuronal maturation through the expression of different late-stage neuronal markers. Using similar two-phase approach, another study first developed neurospheres in a commercial serum-free medium containing BDNF and neurotrophin-3 (NT-3). Then, cells were transferred to Neurocult™ differentiation medium with EGF/bFGF. The obtained cells showed mature neuronal proteins and specific dopaminergic markers. Although this study employed calcium imaging to prove their undifferentiated DPSCs had functional neurotransmitter receptors, they omitted functional testing for their final differentiated cells (24).

Taking a different approach, Li et al. (25) fabricated heparin-modified gelatin/polycaprolactone nanofiber membrane loaded with vascular endothelial growth factor (VEGF), bone morphogenetic protein 2 (BMP-2), and nerve growth factor (NGF). One week after culturing DPSCs, cells started expressing mature neuronal proteins. The authors further validated their model *in vivo* by loading the harvested membrane into prepared root canals of extracted human premolars and implanting them into subcutaneous pockets in mice. After 12 weeks, successfully regenerated dental pulp-like tissues were obtained with confirmed neural and vascular ingrowth. This study revealed that the use of 3D scaffold combined with growth factors can accelerate neurogenic differentiation by activating multiple regenerative pathways.

While supplementation of growth factors is considered a classical method for neural induction, it still has significant limitations. Growth factors are normally applied in a soluble form within the standard liquid media, which causes fast degradation and limited effectiveness. This calls for constant replenishing, leading to an extended induction period, and higher cost (26). In fact, the 3D scaffold utilized by Li et al. (25), which physically immobilized the growth factors, could solve this problem.

Additionally, while growth factor-based protocols successfully upregulated structural markers for mature neurons, some of these markers are inherently present in non-differentiated DSCs (6). Relying solely on structural markers is therefore insufficient, yet, none of the reviewed growth factor-based studies provided terminal functional validation for their workable protocol via electrophysiological assays.

### 3.2 Small molecule-mediated neurogenic induction

To overcome the high costs and rapid degradation associated with recombinant growth factors, researchers have shifted toward neurogenic small molecules and chemical inducers. Unlike growth factors that rely on surface receptor activation, cell-permeable small molecules could directly alter the gene expression of stem cells by targeted intracellular signaling modulation and epigenetic modifications (27). This approach can enhance the conversion efficiency during neuronal differentiation, while providing greater stability, affordability, in addition to shorter induction period (28). Table 2 summarizes the small molecules used in the reviewed studies, their classes, and physiological effects.

**Table 2:** Commonly used small molecules and their physiological effects.

Small molecule	Class	Function	Physiological effects	Reference
Tricyclodecane-9-yl-xanthogenate (D609)	Signaling pathway modulator	PC-PLC inhibitor	Induces cholinergic neuronal differentiation while suppressing non-neural lineages.	(30, 31)
Melatonin	Signaling pathway modulator	MT1 receptor activator	Triggers neurite outgrowth and drives dopaminergic/ neuronal differentiation by increasing TH and decreasing GFAP expression.	(29)
Retinoic acid (RA)	Signaling pathway modulator	RAR/RXR activator	Drives early neural specification, neurite outgrowth, and structural maturation.	(4)
Resveratrol (RSV)	Signaling pathway modulator	TrkA/SIRT1 activator → upregulates CREB	Enhances neuronal differentiation and neuroprotection.	(46)
β-mercaptoethanol (BME)	Metabolic Modulator	Thiol Reducer/ Antioxidant: increase intracellular glutathione → increase ChAT activity → altered neurite outgrowth	Initially claimed to act as a neural pre-inducer that temporarily inhibits apoptosis and upregulates early neuroplastic markers. Subsequent studies showed that the observed neuronal morphology are likely false-positive artifact of stress-induced cytoskeletal retraction.	(31, 38)
Butylated hydroxyanisole (BHA)	Metabolic Modulator	Antioxidant	Protects cells from the massive oxidative stress during differentiation. Later investigations showed that it may causes stress-induced cell shrinkage that creates a reversible, artifactual neuronal morphology, resulting in cell death.	(38)
CHIR99021 (CHIR)	Signaling pathway modulator	GSK-3β inhibitor → Wnt activator	Increases the production of dopaminergic progenitor cells.	(55)
Ascorbic acid 2-phosphate (AAP)	Epigenetic proteins modulator	Histone demethylase activator	Modifies cellular baseline identity by inducing it into a more plastic state to facilitate cell fate transition.	(28)
Valproic acid (VPA)	Epigenetic proteins modulator	Histone deacetylase inhibitor		(27, 53)
GO6983	Signaling pathway modulator	PKC inhibitor		(53)
5-azacytidine (5-AZA)	Epigenetic proteins modulator	DNMT inhibitor		(28, 41)
Y-27632	Signaling pathway modulator	ROCK inhibitor	Facilitates survival of neural lineage cells and promote neurite outgrowth.	(27, 53)
SP600125	Signaling pathway modulator	JNK inhibitor	Facilitates neural reprogramming.	(27, 28)
Forskolin (FSK)	Signaling pathway modulator	Adenylate cyclase activator → increase cAMP and CREB	Regulates neuronal specification and promote axonal regeneration.	(51)
Repsox	Signaling pathway modulator	TGF-β type 1 receptor inhibitor	Enhances neurite outgrowth and cell elongation.	(53)
Dibutyryl cyclic adenosine monophosphate (Db-cAMP)	Signaling pathway modulator	Secondary messenger analog → increase cAMP and CREB	Regulates neuronal specification and promote axonal regeneration.	(28)
Ergothioneine (ERGO)	Signaling pathway modulator	OCTN1 transporter substrate	Acts as a potent BBB-permeable neurogenic enhancer. By actively utilizing the OCTN1 transporter to enter the cell, it drives ectomesenchyme cells to commit to a neuronal lineage.	(48)
Noggin^a^	Recombinant protein antagonist	BMP ligand antagonist → SMAD inhibitor	Inhibits stem cells from adopting non-neural alternative fates, promoting their commitment to a neuronal lineage.	(55)
Dorsomorphin (DM)	Signaling pathway modulator	BMP type 1 receptor inhibitor → SMAD inhibitor		(27)
SB431542	Signaling pathway modulator	TGF-β type 1 receptor inhibitor → SMAD inhibitor		(43)
LDN193189	Signaling pathway modulator	BMP type 1 receptor inhibitor→ SMAD inhibitor		(43)

This table outlines the various small molecules and chemical compounds utilized in neurogenic differentiation protocols, categorized by their primary pharmacological class (Signaling pathway modulators, Metabolic modulators, and Epigenetic protein modulators). It details the specific mechanistic function of each molecule alongside its physiological effects on cellular behavior, including neural specification, neurite outgrowth, and cell survival.

BBB, blood brain barrier; BMP, bone morphogenetic protein; cAMP, cyclic adenosine monophosphate; ChAT, choline acetyltransferase; CREB, cAMP-responsive element-binding protein; DNMT, DNA methyltransferase; GFAP, glial fibrillary acidic protein; GLI, glioma-associated oncogene homolog; GSK-3β, glycogen synthase kinase-3β; JNK, c-Jun N-terminal kinase; MT1, melatonin receptor type 1; OCTN1, organic cation transporter N1; PCL-C, phosphatidylcholine-specific phospholipase C; PKC, protein kinase C; Ptch, patched receptor; RAR, retinoic acid receptor; ROCK, Rho-associated coiled-coil-containing protein kinase; RXR, retinoid X receptor; SIRT1, sirtuin 1; SMAD, small mothers against decapentaplegic; Smo, smoothened; TGF-β, transforming growth factor-β; TH, tyrosine hydroxylase; TrkA, tropomyosin receptor kinase; Wnt, Wingless-related integration site**.**

a Noggin is a recombinant protein morphogen, not a traditional small molecule. It is included in this table due to its comparable role as a targeted BMP/SMAD pathway inhibitor.

Multiple investigations utilized this induction strategy, mainly culturing DPSCs as 2D adherent monolayers. One study investigated dopaminergic differentiation using melatonin (29), a neurohormone that plays numerous roles in various physiological processes, such as regulation of blood pressure, memory formation, oxidative stress, cell proliferation and differentiation. In this study, DPSCs were cultured in a serum-free commercial dopaminergic medium supplemented with melatonin for 21 days, ultimately confirming the expression of mature neuronal and dopaminergic markers through immunocytochemistry (ICC) and western blotting.

Using a much faster protocol, two other studies targeted cholinergic differentiation in only 3-4 days by applying tricyclodecane-9-yl-xanthogenate (D609), a specific inhibitor of phosphatidylcholine-specific phospholipase C (PC-PLC) (30, 31). Inhibiting this enzyme can upregulate functional neuron-specific genes and accelerate cholinergic maturation (30). Both studies reported the expression of cholinergic proteins. Also, both confirmed a significant increase in acetylcholine concentrations in their culture supernatant compared to undifferentiated counterparts by using different neurotransmitter release assays.

One of these teams advanced further by transplanting the *in vitro*-differentiated cholinergic neurons into an experimental rat model with sciatic nerve defects (30). After eight weeks *in vivo*, the rats showed motor nerve regeneration. This recovery was demonstrated through improved behavioral activity in the open field test, also structurally confirmed via the immunohistochemistry tracking of regenerating axonal fibers. At last, the major advantage of this D609 protocol is its brief induction period, which makes it attractive for clinical application.

However, to classify a differentiated cell as a true neuron, it must fulfill three core criteria: exhibiting a structural neuronal phenotype, producing neuron-specific gene transcripts, and displaying functional electrophysiological behavior such as synaptic transmission and the generation of action potentials (32). In fact, combining both voltage- and current-clamp modes of patch-clamp is widely regarded as the gold standard for *in vitro* functional validation. Hence, despite these positive outcomes, the aforementioned 2D monolayer studies (29–31) lacked patch-clamp electrophysiological assay, leaving the cells' ability to generate action potentials unconfirmed.

Among the four small molecules-based studies, only one applied the 3D culture technique (33). Firstly, dentospheres were developed by growing DPSCs in a serum-free NeuroCult proliferation medium supplemented with heparin, EGF, and bFGF for three weeks. To initiate differentiation, these spheres were then transferred into a commercial neural differentiation medium supplemented with retinoic acid (RA), and 1 h pulses of KCl were introduced every two days, starting on the seventh day of induction. RA is one of the most vital morphogens involved in the early developmental phases of central nervous system, where it initiates neurogenesis (34, 35), whereas the recurrent KCl depolarizations aid in inhibiting proliferation and promoting neurite outgrowth (36). After 21 days, this protocol stimulated the expression of mature structural, synaptic, and GABAergic markers.

Beyond phenotype validation, this study further assessed electrophysiological characteristics of the differentiated cells. While the cells exhibited voltage-dependent sodium and potassium currents, they were unable to generate action potentials. To overcome this limitation, the researchers prolonged the induction period up to 60 days, after which, they reported a single action potential was generated during each depolarization. Moreover, voltage- and current-clamp recordings demonstrated that neuro-differentiated DPSCs displayed spontaneous activity in the absence of electrical stimulation. Yet, even with this prolonged induction, the cells failed to generate repetitive action potentials upon stimulation, which is one of the characteristics of mature neurons. Furthermore, the study relied on *in vitro* validation, leaving the *in vivo* survival and network integration abilities of these cells unverified.

Although directing differentiation with small molecules has distinct benefits, it is important to note that particular chemical inducers may cause false-positive neural differentiation, especially when utilized without growth factors. For example, the Woodbury induction protocol (37) that uses *β*-mercaptoethanol and butylated hydroxyanisole. Succeeding investigations have demonstrated that the rapid morphological changes seen after such protocols are stress-induced cytoskeletal collapse rather than actual neuronal differentiation. Besides, these artifactual variations are unstable and readily reversible upon removal of the toxic induction medium (12, 38). Nevertheless, because these specific chemicals eventually fail to establish complete differentiation, protocols utilizing them as standalone agents did not meet our inclusion criteria and were accordingly excluded from this review as outlined in our methodology.

### 3.3 Combination-mediated neurogenic induction

While the separate use of growth factors and small molecules has demonstrated clear advantages, these isolated approaches often fail to achieve complete and stable functional maturation. Recognizing the toxicity of pure chemical induction, most of the reviewed studies shifted to a mixed approach. Combining these mediators allows the proliferation of initial stem cell population while concomitantly directing the cells to a specified, physiologically active neuronal subtype (12, 39).

However, there is no standardized protocol for the application of these factors. Some studies relied on step-wise, multi-phase protocols, in which, small molecules are used first to erase the cells’ baseline identity and induce a state of plasticity, followed by growth factors to stimulate neuronal maturation. Meanwhile, other studies adopted a single-phase strategy, inducing differentiation with a synergistic cocktail of both mediators simultaneously.

Even with these advanced strategies, a significant gap remains in the literature. Several 2D (13, 40–42) and 3D (35, 43, 44) models depended solely on phenotype profiling without any functional validation. Yet, the induction methods were highly innovative. Attempting to mimic the natural developmental niche, two groups used RA with embryonic rat cerebrospinal fluid (CSF) (35, 40). CSF naturally delivers a mix of neurogenic factors, including transforming growth factor beta (TGF-*β*), insulin-like growth factor (IGF), nerve growth factor (NGF), bFGF, BDNF, and NT-3. To demonstrate the success of this specialized microenvironment, one of these studies (40) specifically highlighted the formation of prominent Nissl bodies within the differentiated cells. However, relying on this structural evidence has limitations. While the presence of prominent Nissl bodies is a recognized feature of mature, metabolically active neurons, Nissl stains broadly bind to rough endoplasmic reticulum, meaning any highly active cell type could yield a visually similar result.

Another team employed a complex three-phase strategy (41). They started by erasing cellular memory with azacytidine, then they triggered depolarization with a high-potassium buffer, and finally drove maturation in a serum-free medium of cyclic adenosine monophosphate (cAMP) elevators and neurotrophins. Five days post-induction, their differentiated cells were shown to express postsynaptic density protein 95 (PSD95). Other researchers combined chemical induction with genetic engineering. One study used lentivirus to overexpress insulin-like growth factor in SCAPs, followed by a two-phase protocol that upregulated both excitatory and inhibitory synaptic markers (42). Taking a 3D approach with the same stem cell source, another group encapsulated SCAPs inside a gelatin methacryl hydrogel that was pre-loaded with BDNF. Then added their induction cocktail of small molecules and neurotrophic factors. They reported that the 3D environment significantly enhanced the neurogenic potential of SCAPs (44).

To produce a specialized auditory spiral ganglion neurons (SGNs), one study implemented a prolonged, stepwise 3D protocol (43). They initially formed neurospheres using EGF/bFGF-containing media, which was supplemented with precise small molecules to block the TGF-*β* and BMP pathways and force early neural specification. BMP4 was then added to drive DPSCs toward an otic lineage that is finally directed to mature by neurotrophic factors. While standard structural markers appeared after 21 days, the specific SGN markers required 32 days to emerge. To support these outcomes, this group used atomic force microscopy to prove that their *in vitro* differentiated SGN has a similar nanomechanical stiffness to the native SGNs. They additionally reported the cells’ ability to physically attract neurites from rat explants *ex vivo*.

In an attempt to overcome the known toxicity of chemical induction, a study utilized resveratrol (RSV) (13), a natural Sirtuin 1 activator known for its neuroprotective properties (45). Following a modified 30 h Woodbury protocol (37), DPSCs were pre-treated with RSV for 12 h before induction. The cells finally had both early and mature structural neuronal proteins. However, depending exclusively on structural markers can be deceiving, as phenotype validation does not always reflect actual neuronal differentiation, and conversely, some functional characteristics can appear before structural maturity. This contradiction was illustrated by a succeeding study applying the same resveratrol-enhanced protocol for SCAPs differentiation (46). Pre-treatment with resveratrol prompted the initial genetic shift toward a neural lineage as demonstrated by the upregulation of the early marker SRY-box transcription factor 1 (SOX1). However, the cells did not show late-stage structural markers, leaving them classified as neural progenitors. Yet, when calcium imaging was conducted, these progenitor cells displayed sudden, functional intracellular calcium oscillations upon KCl depolarization.

It is hard to miss that while these two studies (13, 46) used the same RSV-enhanced induction protocol, they produced conflicting structural outcomes. While the first study upregulated both early and late structural markers, the second study failed to express the late markers. This discrepancy may be related to the Woodbury protocol that is used as baseline or due to the varied cell origin. RSV as a potent natural neurogenic enhancer (47), holds significant potential for DSC neurogenic differentiation, but its true efficacy will only be seen when combined with more reliable induction strategies.

Two other studies employed provoked calcium imaging to confirm active ion channel activity in more mature phenotypes (27, 48). These groups achieved comparable outcomes using different induction periods. One study utilized an extended, two-phase approach, starting by priming the cells with an eight-molecule chemical cocktail and then maturing them with neurotrophic factors (27). After 14 days, they achieved calcium fluxes across all compared oral stem cell lines. The second study used a 30 h ergothioneine improved induction medium (48). Ergothioneine is a natural compound that has valuable effects on neurite outgrowth, neuronal differentiation, and neuronal maturation (49, 50). In addition to calcium imaging, they recorded the expression of synaptic vesicle glycoprotein 2A (SV2A), a key protein that contributes in regulating neurotransmitter release (48).

Moving beyond isolated calcium currents to chemical communication, one study evaluated both mechanisms simultaneously. In addition to confirming positive calcium transients, this group also quantified neurotransmitter release (51). They used a cocktail of growth factors and forskolin to induce dopaminergic neuron differentiation within two weeks. Forskolin can enhance the intracellular cAMP levels, which helps to activate neuronal specification and axonal outgrowth. After induction, the dopamine level was significantly detected in the culture supernatant. Moreover, the development of mature physical synaptic apparatus was verified by scanning and transmission electron microscope.

Taking a similar biochemical approach but focusing on a different neuronal subtype, another study assessed the secreting abilities of differentiated cholinergic neurons (31). The researchers compared three distinct differentiation strategies: a *β*-mercaptoethanol/NGF approach, the previously mentioned small-molecule D609 protocol, and a synergistic combination of bFGF, SHH, RA, and forskolin. While all three methods yielded cells expressing cholinergic-specific proteins and secreted significantly more acetylcholine than undifferentiated controls, the synergistic cocktail (protocol III) produced the best structural and functional outcomes.

Taking functional validation a step further, the following studies crossed the threshold from biochemical analysis to true electrophysiological excitability. One study used patch-clamp recordings strictly in voltage-clamp mode following a sequential induction strategy: RA to prime the cells, and BDNF to drive maturation (4). Electrophysiological data demonstrated upregulation of both voltage-dependent sodium and potassium currents compared to untreated controls, proving that the cells had developed functional ion channels across their membranes. However, these induced currents were notably small.

Another study demonstrated that while a 35-day monolayer protocol generated structural neural markers, the resulting cells completely lacked electrical currents and were bursting instantly under patch-clamp seals (52). On the other hand, when the same group utilized a 3D neurosphere approach combining an initial 8 days, serum-free EGF/bFGF expansion phase with a four-week dibutyryl cAMP/NT-3 maturation phase, the cells acquired functional excitability. Voltage-clamp analysis confirmed the presence of sodium and potassium currents that were reversibly blocked by tetrodotoxin (TTX) and tetraethylammonium (TEA). Furthermore, current-clamp recordings elicited a single electrical action potential upon stimulation.

Comparable functional outcomes were achieved by using a 7 days, small-molecule cocktail induction protocol (53). This cocktail was designed to concurrently erase epigenetic memory and modifying main developmental pathways to stimulate rapid neurogenic commitment of SCAPs.

However, despite reaching this *in vitro* validation, functional limitations appear across both protocols (52, 53). The overall yield of excitable cells remained notably low, with only a small proportion of the tested cells actually showing action potentials. Also, these induced action potentials required electrical stimuli rather than being spontaneous.

Taking the previous strategy (53) a step further, a separate study employed the same small-molecule cocktail and SCAP model to evaluate neuronal regenerative potential *in vivo* (54). Using a scaffold-free cell sheet technique, the researchers transplanted the differentiated cells directly into a rat sciatic nerve defect. Functionally, this *in vivo* transplantation yielded enhanced motor function recovery on walking track test. However, while the implanted cells survived the transplantation, they poorly integrated into the host tissue. The cells formed dense clusters which likely hindered the long-term projection of host nerve fibers and ultimately may have limited the reinnervation of the severed region.

Interestingly, by viewing these two independent papers together (53, 54), this specific induction strategy effectively achieves a complete validation profile: single-cell electrical firing *in vitro* (53), and tissue regeneration *in vivo* (54)*.* However, the outcomes were not optimal.

Representing the peak of combined *in vitro* and *in vivo* validation, one comprehensive study bridged the gap between cellular excitability and functional network integration (55). Following a three-phase sequential strategy, SHEDs were guided into a dopaminergic lineage. First, researchers incorporated Noggin, a targeted inhibitor that suppress competing SMAD-signaling pathways, priming the cells toward an initial neural fate. Next, to promote dopaminergic specification, they employed CHIR99021 to activate the Wingless-related integration site (Wnt) signaling pathway, while immersing the cells in morphogenic factors (SHH and FGF8). Finally, the cells were exposed to a cocktail of neurotrophins to drive terminal maturation. Before proceeding to animal models, they confirmed the cells' electrophysiological maturity using both patch-clamp modes. Utilizing voltage-clamp to record spontaneous currents, and switching to current-clamp to provoke electrical action potentials. However, it must be noted, that their *in vitro* reporting lacked quantification, demonstrating action potential activity using qualitative visual traces alone.

Nevertheless, the true strength of this study appears in its *in vivo* follow-up (55). Upon transplantation into a 6-hydroxydopamine-lesioned parkinsonian rat model, behavioral motor recovery was reported after 16 weeks. The researchers validated this functional recovery using a dual behavioral approach. First, they detected a progressive reduction in apomorphine-induced rotational asymmetry, proving the grafted cells were rebalancing the neurochemical shortage in the lesioned hemisphere. Second, utilizing rotarod latency tests, they demonstrated the restoration of gross motor coordination and balance with time. Importantly, they avoided the structural clustering seen in the previous study (53). Histological analysis confirmed that the transplanted cells sprouted neurites and formed physical synaptic connections with the host neurons. Additionally, the researchers performed *ex vivo* amperometric recordings on the grafted brain slices, proving that the transplanted human cells were actively firing and secreting dopamine directly within the host network.

Even at this advanced stage of functional validation, absolute lineage control remains practically unattainable. Despite targeted pathway manipulation and continuous neurotrophic support, the researchers reported a spontaneous glial differentiation where 20% of the transplanted cells drifted from a neuronal fate into an astrocyte lineage. This highlights the final challenge in stem cell neuro-regeneration, which is off-target differentiation. Moving forward, overcoming this lineage drift may require induction strategies that go beyond extracellular signaling. Future protocols could explore combining small-molecule modulators with targeted genetic regulators, such as specific microRNAs or transcription factors, to suppress alternative pathways and stabilize the neuronal phenotype during terminal maturation. Figure 1 illustrates an optimized combined strategy for DSCs neurogenic differentiation.

**Figure 1:**
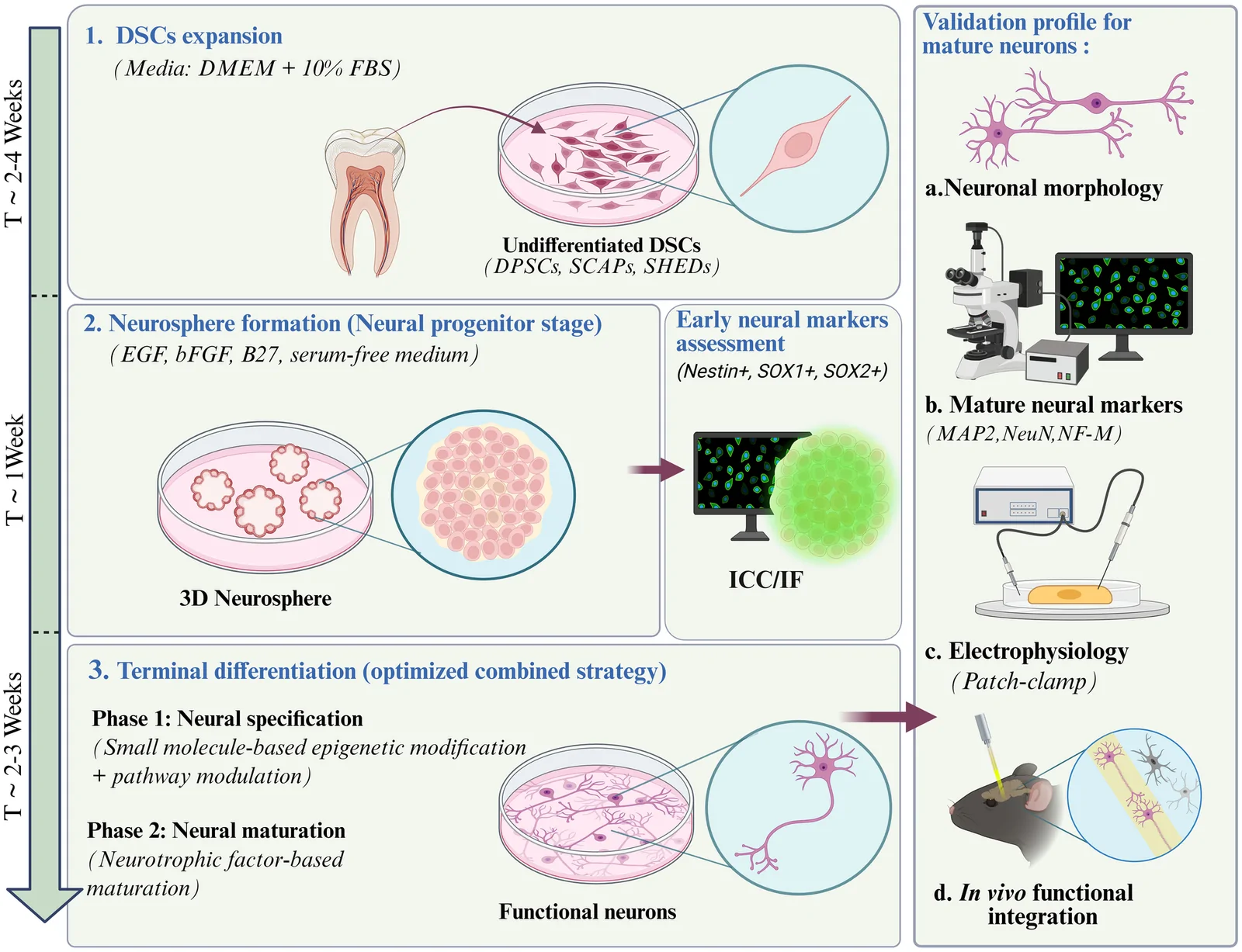
A proposed optimized framework for the neurogenic differentiation of dental stem cells. The schematic outlines a chronological three-step process. Step 1 involves the initial expansion of undifferentiated DSCs (DPSCs, SCAPs, or SHEDs) in standard media. Step 2 transitions the cells into 3D neurospheres to reach the neural progenitor stage, allowing for the assessment of early neural markers such as Nestin, SOX1, and SOX2. Step 3 drives terminal differentiation through an optimized combined strategy divided into two phases: neural specification via small molecule-based epigenetic and pathway modulation, followed by neural maturation driven by neurotrophic factors. The right section illustrates multi-modal validation profile required to confirm mature functional neurons, which involves the assessment of neuronal morphology, detection of mature neural markers (MAP2, NeuN, NF-M), electrophysiological functional testing via patch-clamp recording, and successful *in vivo* functional integration. B27, B27 supplement; bFGF, basic fibroblast growth factor; DMEM, Dulbecco's Modified Eagle Medium; DPSCs, dental pulp stem cells; DSCs, dental stem cells; EGF, epidermal growth factor; FBS, fetal bovine serum; ICC/IF, immunocytochemistry/immunofluorescence; MAP2, microtubule-associated protein 2; NeuN, neuronal nuclei; NF-M, neurofilament medium; SCAPs, stem cells from the apical papilla; SHEDs, stem cells from human exfoliated deciduous teeth; SOX1, SRY-box transcription factor 1; SOX2, SRY-box transcription factor 2. Created in BioRender. Alsultani, M. (2026).

## 4 Limitations

Although a defined search strategy was utilized to identify studies from the past decade, the primary search was restricted to a single biomedical database (PubMed) and limited to English-language publications. As a result, relevant articles indexed in other databases or published in other languages may not have been retrieved. Despite efforts to manually add studies found through secondary cross-referencing, a subset of related literature may have been unintentionally excluded.

In addition, the reviewed studies demonstrated considerable methodological variation. Differences in donor age and health, the types of dental stem cells used and their passage number, basal media, and induction timelines make it challenging to directly compare the efficacy of differentiation protocols.

Finally, as highlighted throughout the review, because the majority of studies relied on structural markers rather than *in vitro* functional testing or *in vivo* integration, it remains difficult to conclude which protocol yields fully functional neurons.

## 5 Conclusions and future directions

Despite the wide variety of neurogenic induction protocols developed for dental stem cells (specifically DPSCs and SCAPs) over the past decade, definitive, functional neurogenesis remains challenging *in vitro*. This is possibly related to two critical flaws noted in the current literature: a lack of standardized neurogenic induction strategies and a heavy dependence on structural morphology and biochemical marker expression instead of electrophysiological validation.

Nevertheless, utilizing a step-wise combination of small molecules and neurotrophic growth factors emerges as a promising neurogenic induction strategy. While isolated chemical induction risks false-positive morphological artifacts, and growth factor application is limited by rapid degradation, this multi-phase combined approach helps lineage-specific commitment while preserving cellular viability. Furthermore, given the potential biological benefits of 3D neurosphere cultures and serum-free media, future studies can apply these combined protocols within these optimized environments. Finally, future research attention should be directed toward true cellular excitability, such as using patch-clamp electrophysiology, and *in vivo* network integration. These can serve as the essential foundation required to eventually translate *in vitro* models into viable neuroregenerative therapies.
